# Protein rethreading: A novel approach to protein design

**DOI:** 10.1038/srep26847

**Published:** 2016-05-27

**Authors:** Sayeh Agah, Sandra Poulos, Austin Yu, Iga Kucharska, Salem Faham

**Affiliations:** 1 Department of Molecular Physiology and Biological Physics, University of Virginia School of Medicine, Charlottesville, Virginia, 22903, United States

## Abstract

Protein engineering is an important tool for the design of proteins with novel and desirable features. Templates from the protein databank (PDB) are often used as initial models that can be modified to introduce new properties. We examine whether it is possible to reconnect a protein in a manner that generates a new topology yet preserves its structural integrity. Here, we describe the rethreading of dihydrofolate reductase (DHFR) from *E. coli* (wtDHFR). The rethreading process involved the removal of three native loops, and the introduction of three new loops with alternate connections. The structure of the rethreaded DHFR (rDHFR-1) was determined to 1.6 Å, demonstrating the success of the rethreading process. Both wtDHFR and rDHFR-1 exhibited similar affinities towards methotrexate. However, rDHFR-1 showed no reducing activity towards dihydrofolate, and exhibited about ~6-fold lower affinity towards NADPH than wtDHFR. This work demonstrates that protein rethreading can be a powerful tool for the design of a large array of proteins with novel structures and topologies, and that by careful rearrangement of a protein sequence, the sequence to structure relationship can be expanded substantially.

Substantial progress has been made in the field of protein design mainly by relying on computational methods^1^. For example, with the rosetta software, it has been possible to design a novel protein fold^2^, a 2D lattice^3^, self-assembling nano particles^4^, and novel enzymes^5^,^6^. Other computational methods have relied on templates from the protein data bank (PDB) that can be optimized^7^. It has also been possible to computationally design a membrane protein^8^. Here we introduce an easy to use protein design method that is not computationally heavy, yet can be a powerful approach for the generation of many novel protein structures and topologies.

Similar to circular permutations, here we connect regions that are close in three dimensional (3D) space, but distant in sequence. Circular permutations have demonstrated that it is possible to connect the N and C termini of proteins with minimal changes to their structures^9^,^10^. It has been shown that circular permutations can generate valuable features such as improved protein thermo-stability, enhanced catalytic activity^11^,^12^, reduced proteolytic susceptibility^13^, and altered substrate binding^11^,^14^, in addition to the development of novel biocatalysts^15^,^16^ and biosensors^17^. One constraint with circular permutations is that it can only be carried out on proteins with proximal N and C termini. The N and C termini may appear unique in having free amino and carboxyl groups. However, free amino and carboxyl groups can be introduced by breaking any peptide bond. Indeed, a variety of new N and C termini can be artificially introduced. If these newly introduced termini can be reconnected in a manner similar to what’s performed in circular permutations, then a vast array of structural rearrangements may be possible. Here, we examine whether it is possible to rearrange a protein structure with newly introduced amino and carboxyl groups. This process can be described as “protein rethreading”, where instead of threading the protein according to its linear sequence^18^,^19^, the polypeptide chain makes alternate connections at suitable junctions that may be available in its three dimensional (3D) structure. Protein structures reveal that residues can be very distant in sequence, but in close proximity in 3D space. These spatially proximal residues offer sites where the path of the peptide chain may be altered. A simple approach to accomplish protein rethreading, is to keep the secondary structural elements intact, and to swap some of the connecting loops. This type of structural rearrangement has been observed in naturally occurring proteins, and has been described as a form of multiple loop permutations (MLP)^20^.

For the rethreading process, we selected dihydrofolate reductase from *Escherichia coli* (wtDHFR), which has been shown to tolerate a variety of circular permutations^21^. DHFR (EC 1.5.1.3) is a ubiquitous enzyme that utilizes the reduced form of nicotinamide adenine dinucleotide phosphate (NADPH) to catalyze the reduction of 7,8-dihydrofolate (DHF) to 5,6,7,8-tetrahydrofolate^22^. DHFR is an essential enzyme, and a clinically relevant drug target that is inhibited by the antibacterial compounds trimethoprim and methotrexate. In our case, the original termini of DHFR were left undisturbed. We did not perform circular permutation, nor did we connect either of the original termini to a newly introduced one. In a protein rethreading process, a number of native loops need to be removed and a number of new loops introduced. MLP, which has been described computationally^20^, can be considered as a form of protein rethreading. Similarly, non-sequential structural analogs have also been identified computationally^23^. However, protein rethreading has never been demonstrated experimentally. Protein rethreading can be a challenging process. An apparent challenge is that all structural rearrangements have to be carried out successfully simultaneously to prevent the fragmentation of the polypeptide chain. Thus, it is not possible to experimentally determine which removed loop or newly introduced one is disrupting the protein structure, in case the rethreaded protein is problematic. If any of the removed loops are essential, or any of newly introduced loops are not suitable, then the new protein may not fold properly, or may be unstable. As a result, there is a good chance that the rethreaded protein may not behave as desired. We have determined the structure of rethreaded DHFR (rDHFR-1) to 1.60 Å, and carried out the biochemical characterization of the rethreaded protein. We have also tested if protein structure prediction programs were able to predict the structure of rDHFR-1.

## Methods

### Protein design

Our structural rearrangement of DHFR involved the simultaneous removal of three loops and the introduction of three alternate connections. It is not possible to test the change of one loop at a time, because that would lead to fragmentation of the polypeptide chain (Supplemental Figure 1). Connecting two distant regions with a new loop requires the breakage of two peptide bonds, as a result the protein will be broken into two polypeptide chains that are not linked as shown in Supplemental Figure S1. In order to maintain the protein as a single polypeptide chain a third bond needs to be broken, and a total of three loops introduced. Thus, three loops need to be exchanged in a single step, in order to maintain the protein as a single polypeptide chain.

**Figure 1:**
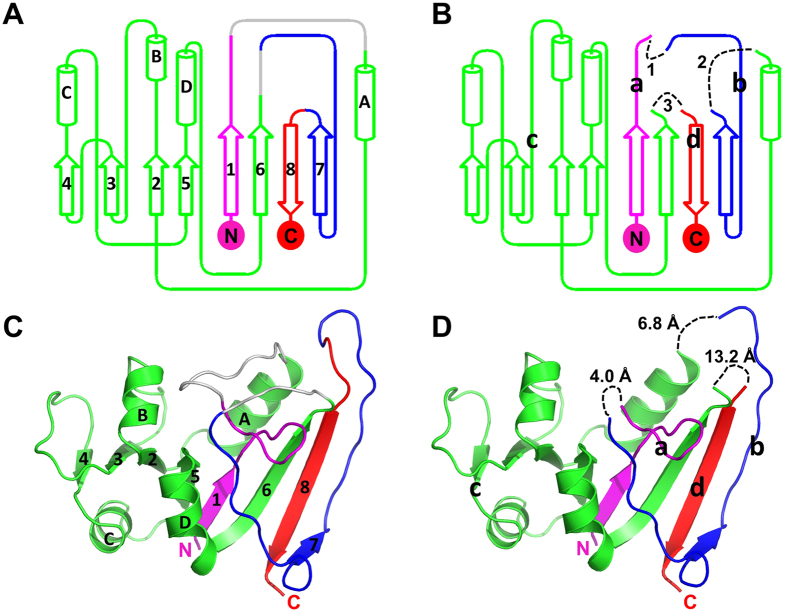
(**A**) topology of the native *E. coli* DHFR, and its (**C**) three dimensional structure. (**B**,**D**) show the three loops that are removed in the topology diagram and in the three dimensional structure, respectively. The three new connections are shown as dashed lines in black (**B**,**D**). The numbering for the three loops is shown in (**B**). The four fragments [a–d] are labeled in (**B**,**D**). In all four panels, fragment [a] (residues 1–15) is colored in magenta, [b] in blue (residues 122–147), [c] in green (residues 25–118), and [d] (residues 149–159) in red. Removed residues are shown in gray. The distances between the termini of the fragments are shown in (**D**), and are based on wtDHFR structure (pdb code 1RX9). These distances were measured from the carbonyl carbon to the amide nitrogen. The distance between the [a,b] fragments is 4.0 Å (residue Gly15 to Asp122); the distance between the [b,c] fragments is 6.8 Å (residue Asn147 to Pro25); and the distance between the [c,d] fragments is 13.2 Å (residue Glu118 to Ser148).

**Figure 2:**
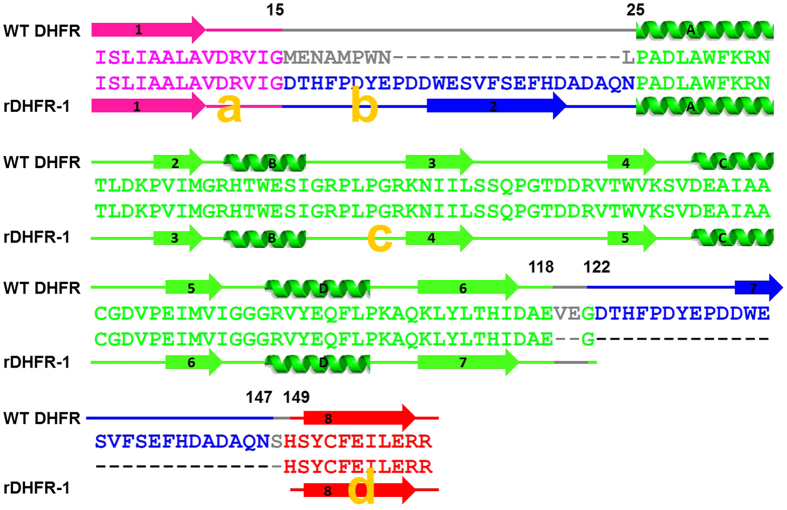
Sequence alignment between the wild type *E. coli* DHFR and the rethreaded DHFR. The residue numbers at the termini of the fragments are shown for the native protein. The secondary structural elements are labeled. The four fragments are labeled in yellow and are colored using the same scheme as in Fig. 1.

The rethreading process carried out can be described as breaking the protein into four fragments (a–d) that were then stitched back together using alternate connections (Fig. 1). The four fragments are composed of the following residues: [a] (1–15), [b] (122–147), [c] (25–118), and [d] (149–159). These four fragments were stitched back together in the order shown (a –>b –>c –>d), such that residue 15 was connected to 122, 147 connected to 25, and 118–149. The wild type protein is connected in the following order (a –c –b –>d) (Fig. 2).

The introduction of new residues was avoided to minimize the modifications to the native sequence. Therefore, to link the fragments we relied on the residues that were already part of the native sequence at the incision sites. The distances between the termini of the connected fragments in the wtDHFR (PDB code 1RX9)^24^ protein are: 4.0 Å, 6.8 Å, and 13.2 Å. The distances were measured from the carbonyl carbon to the amide nitrogen. These distances are longer than a single peptide bond; however, the incisions were made within loop regions, such that there were a number of loop residues remaining on both sides of the broken bond. These loop residues can be expected to be flexible, thus they can adjust to a new conformation bridging the distance. We have observed this to be true in a previous heterodimeric fusion^25^. Although, the termini of the flagellar assembly proteins (FliS and FliC) are ~14 Å apart, it was possible to fuse the termini with just a two amino acids linker with minimal changes to the structure of the heterodimeric complex.

The requirement for breaking 3 bonds and forming 3 new connections establishes restrictions on where in the structure rethreading can be carried out. By visual inspection of the protein structure we identified suitable points in the protein that can be used to perform rethreading. The distances between the incision sites were used to determine the minimum number of residues needed to make the new connections. The first incision was made at positions 15 and 25, removing residues (16–24), and leaving behind 6 preceding loop residues and only one subsequent loop residue (Pro-25). The second incision was made at positions 118 and 122, leaving behind 4 preceding loop residues and 10 subsequent loop residues. The third incision was made at positions 147 and 149, leaving behind 13 preceding loop residues and 2 subsequent loop residues (Fig. 2). As a result, all three new connections had sufficient loop residues left behind to form the new loops. For example, for the first new connection, 6 loop residues remain on the [a] fragment, and 10 loop residues remain on the [b] fragment. These 16 residues are more than sufficient to bridge the 4 Å distance between the two incision points (Figs 1 and 2). The loop residues are expected to be flexible and able to adopt the required changes^25^. The number of loop residues varies depending on which model is used for this analysis. In this case, 1RX9^24^ was used for these measurements.

### Cloning, protein expression, and purification

The rethreaded DHFR sequence, referred to as rDHFR-1, was prepared using gene synthesis by Genewiz. The synthesized gene was provided in pUC57 vector and was sub cloned into a pBAD vector using NcoI and XhoI cut sites. A hexa-histidine tag was designed at the C-terminus end. The protein was over-expressed in TOP10 *E. coli* cells in LB containing 100 μg/mL ampicillin. The cells were grown at 37 °C, until the OD_600_ reached 0.7. The temperature was then reduced to 28 °C, and the cells were induced for 4 h with 0.02% L-arabinose. For the NMR experiments, rDHFR-1 was cloned into a pET-28 vector, expressed in BL21, and 0.5 mM IPTG was used for induction. To obtain uniformly ^15^N-labeled protein, minimal medium (containing 1 g/liter ^15^N (98%)-labeled (NH_4_)_2_SO_4_, (Cambridge Isotope Laboratories)) was used instead of LB medium. The cell pellets were resuspended in 50 mM Tris, pH 8.0, 150 mM NaCl, 5% glycerol, 2 mM β-mercaptoethanol (BME). The cell suspension was disrupted by several passes through a microfluidizer, and the cell extracts were centrifuged in Beckman JA-2αotor for 30 min at 14,000 rpm. The protein was purified using a Ni-NTA affinity resin that was pre-equilibrated with (50 mM Tris pH 8.0, 150 mM NaCl, 5% glycerol). The resin was washed in subsequent steps with same buffer containing 2 mM BME and increasing imidazole concentrations (10–50 mM). The protein was eluted in 50 mM Tris pH8.0, 150 mM NaCl, 5% glycerol, 2 mM BME, and 250 mM imidazole. The eluted protein was further purified on a Superdex 200 10/300 GL size exclusion column (SEC) (GE Healthcare). The column was pre-equilibrated with buffer containing 50 mM Tris pH 8.0, 150 mM NaCl, 2 mM BME. The protein (rDHFR-1) was concentrated to ~10–20 mg/mL using a 10 kDa Amicon Ultra-4 (Millipore). wtDHFR coding sequence was amplified using the appropriate primers and *E. coli* genomic DNA as a template. The purification protocol for wtDHFR was the same as what described for rDHFR-1.

For the NADPH and Methotrexate binding experiments, a series of dialysis steps were performed to remove any residual bound NADPH after protein purification. The dialysis was performed on both wtDHFR and rDHFR-1. All dialysis steps were carried out at 4 °C for at least 12 hours. First, we dialyzed both proteins against 4 M Urea, 50 mM Tris pH 8.0, 150 mM NaCl, 2 mM ADP, and 2 mM BME. Next, the proteins were dialyzed against 2 M Urea, 50 mM Tris pH 8.0, 150 mM NaCl, 2 mM ADP, and 2 mM BME. The final dialysis step was performed against 25 mM sodium phosphate pH 6.5, 150 mM NaCl, 2 mM BME. Removal of the residual bound NADPH was confirmed by fluorescence spectroscopy with excitation and emission wavelengths at 340 nm and 465 nm respectively.

### Isothermal titration calorimetry

Isothermal titration calorimetry (ITC) measurements were carried out on a VP-ITC 200 microcalorimeter (Microcal, Northampton, MA, USA). All samples were dialyzed against the ITC buffer (25 mM sodium phosphate pH 6.5, and 150 mM sodium chloride) and degassed for 10 minutes before titration. The sample cell was loaded with either 70 μM rDHFR-1 or 50 μM wild type DHFR, and individually titrated with NADPH at a concentration of 1 mM for rDHFR-1 or 500 μM for wtDHFR. Experiments were performed at least in duplicate using the following parameters: temperature, 21 °C; reference power, 10 μcal/s; spacing between injections, 200 s, and the agitation speed 1000 rpm. After the addition of an initial aliquot of 0.5 μL, 19 aliquots of 2 μL of the syringe solution was delivered for rDHFR-1, and 22 aliquots of 1.7 μL was delivered for wtDHFR. Data were analyzed using Origin 7 software provided by the manufacturer with curves fitted to a one set of sites model.

### Fluorescence polarization

One micromolar fluorescein methotrexate triammonium salt (Molecular Probes^TM^) was incubated with increasing concentrations of purified either wtDHFR or rDHFR-1 (0.1–60 μM) in a 25 μL reaction mixture in a 384 well flat bottom plate (Corning, NY). The assay buffer contained 25 mM sodium phosphate pH 6.5, 150 mM NaCl. Fluorescence measurements were taken on a SpectraMax M5 Multi-Mode Microplate Reader (Molecular Devices) with excitation and emission filters at 494 nm and 521 nm, respectively. The *K*_d_ value was obtained by fitting the data in OriginPro 7.5 using the equation:


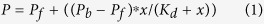


where*, x* is the protein concentration, *P* is the fluorescence polarization (FP) signal measured, and *P*_b_ and *P*_f_ are the fractions of protein bound and free at each point, respectively.

### Activity assay

We tested the activity of both wtDHFR and rDHFR-1 by measuring the loss of absorbance of the NADPH molecule at 340 nm. rDHFR-1 was tested at a 10-fold higher concentration that wtDHFR. 100 nM wtDHFR and 1 μM rDHFR-1 were incubated with 100 μM DHF and 5 mM NADPH in the assay buffer (25 mM sodium phosphate pH 6.5, 150 mM NaCl), and the loss of NADPH was monitored over 15 minutes at 340 nm in a SpectraMax M5 Multi-Mode Microplate Reader (Molecular Devices) (Supplemental Figure S2).

### NMR Spectroscopy

^15^N-rDHFR-1 was expressed and purified as described in *cloning, protein expression, and purification* section. After the purification the protein was dialyzed against a buffer containing 25 mM NaPO_4_ pH 6.0, 50 mM KCl, 0.05% NaN_3_. The final NMR samples were concentrated to 0.25 mM rDHFR-1 and supplemented with 5% D_2_O.

^15^N-^1^H TROSY spectra of rDHFR-1 were recorded at 25 °C on a Bruker Avance III 800 spectrometer equipped with a triple-resonance cryoprobe. Bruker Topspin software version 2.1.6 was used to collect all the NMR experiments. NMR data were processed with NMRPipe and analyzed with Sparky software (Goddard, T.D. and Kneller, D.G. University of California San Francisco).

### Protein crystallization, data collection and structure determination

The protein was crystallized using hanging drop vapor diffusion method at 4 °C. 2 μl of the protein solution and 1 μl reservoir were mixed and allowed to equilibrate. The reservoir solution contained 1.0 M LiCl_2_, 0.1 M Sodium acetate, and 30% PEG 6000. The protein solution contained 9 mg/ml protein, 20 mM tris pH 8.0, 50 mM NaCl, and 10 mM adenosine diphosphate. The crystals were flash cooled at 100 °K without the addition of a cryo protectant since the crystallization condition had a high concentration of PEG. Data collection was carried out at the 22-ID beamline at the Advanced Photon Source (Argonne National Laboratory). The data was processed and scaled using the HKL2000 package^26^. Molecular replacement was carried out using the program Phaser^27^ with native DHFR (PDB code: 2ANO)^28^ as a search model. Refinement was performed using the program REFMAC^29^, and model building was performed using the program Coot^30^.The molecular graphics figures were prepared using the program Pymol (www.pymol.com). Protein topology diagrams were prepared with TopDraw^31^. Data collection and refinement statistics are shown in Table 1. The data was complete to 1.7 Å, but significant data was measured to higher resolutions, and the protein was refined to 1.6 Å (Supplemental Table S1).

### Structural analysis

Prediction of the structure of rDHFR-1 was tested using the programs Phyre2^32^, I-TASSER^33^, Muster^34^, Raptor-X^35^, and the Robetta server^36^. Selection of incision sites were based on the wtDHFR structure (PDB code 2ANO)^28^, which bound both an NADPH and an inhibitor molecule. In some cases, the loops were trimmed but no new residues were added. As a result, the rethreaded protein is 12 amino acids shorter than the wild type *E. coli* protein. The trimmed residues are shown in gray in Figs 1 and 2. The reported distance measurements for the incision sites are from the carbonyl oxygen to the amide nitrogen of the connected fragments. The comparison between rDHFR-1 and wtDHFR was done after superimposing the structures in coot, using the chain A of rDHFR-1 and 1RX9 (pdb code)^24^ as a model for wtDHFR. The NADPH molecule from the A chain of rDHFR-1 is compared with two wtDHFR structures (pdb codes 1RX9 and 4P66)^24^,^37^, with similar results.

### Accession numbers

Coordinates and structure factors have been deposited in the Protein Data Bank (PDB) with the accession number 5DXV.

## Results

### Protein characterization

**Figure 3:**
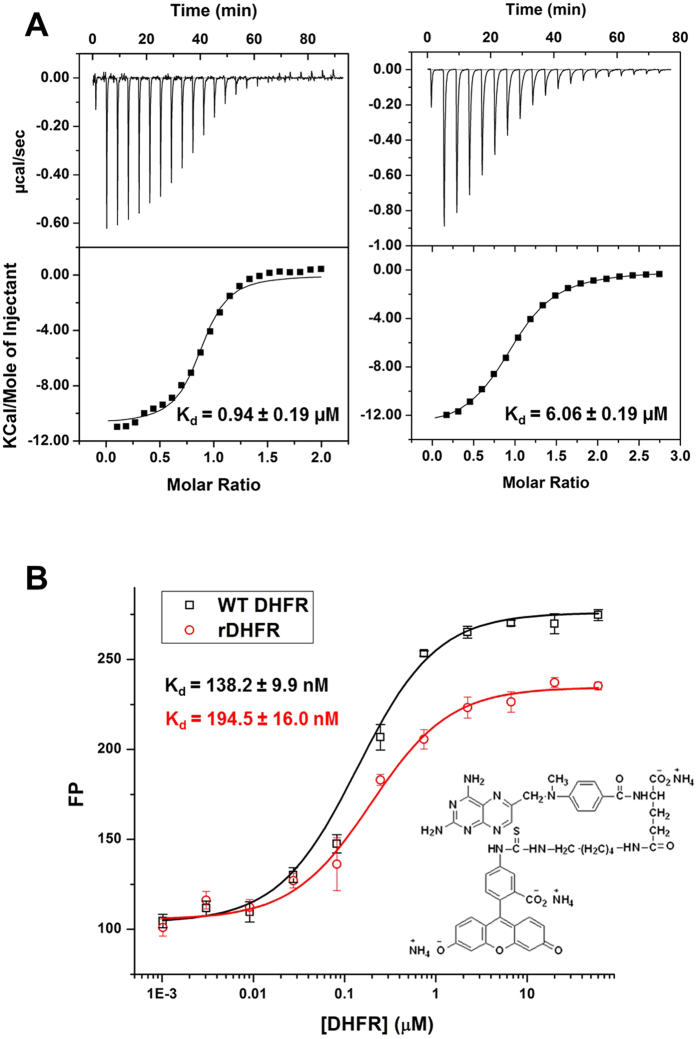
NADPH (**A**) and methotrexate (**B**) binding to wtDHFR and rDHFR-1. (**A**) Isothermal titration calorimetry measurements for NADPH binding to wtDHFR (left panel) show a K_d_ of 0.94 ± 0.19 μM, and to rDHFR-1 (right panel) show a K_d_ of 6.06 ± 0.19 μM. (**B**) Fluorescence polarization show the binding of methotrexate fluorescein to wtDHFR (black line), and to rDHFR-1 (red line). The structure of methotrexate fluorescein is shown in the inset. The X-axis is shown on a logarithmic scale.

**Figure 4:**
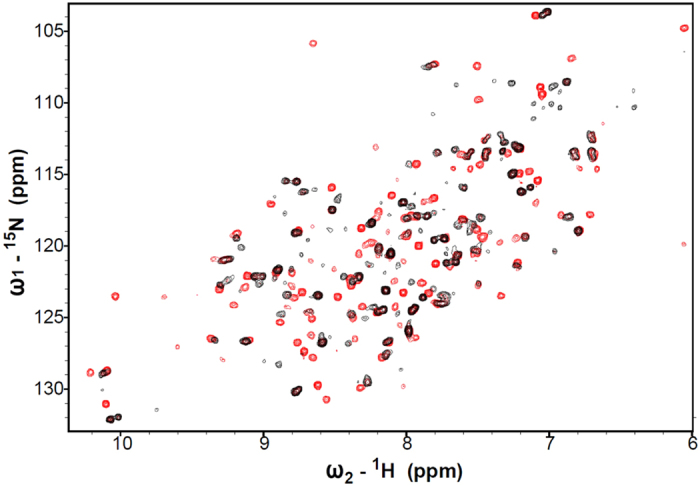
^15^N-^1^H TROSY spectrum of ^15^N-labeled rDHFR-1 (black), overlaid onto the spectrum of ^15^N-labeled rDHFR-1 in the presence of 10-fold molar excess of NADPH (red)

The rDHFR-1 protein eluted as a monomer on the SEC column. It was possible to concentrate the protein to high levels (at least 20 mg/ml), indicating that the protein does not aggregate easily. Binding affinity towards NADPH and methotrexate were measured and compared to the wtDHFR. NADPH is required for the DHFR activity, and methotrexate is a known DHFR inhibitor^38^,^39^. A series of dialysis steps, described in the methods section, was performed to remove any potentially bound NADPH after the purification. NADPH binding to rDHFR-1 and wtDHFR was carried out using isothermal titration calorimetry (ITC). NADPH exhibited a ∼6-fold lower affinity towards rDHFR-1 (K_d_ = 6.06 ± 0.19 μM) compared to wtDHFR (K_d_ 0.94 ± 0.19 μM) (Fig. 3A). Methotrexate binding was measured by fluorescence polarization using a methotrexate analog labeled with fluorescein (Fig. 3B). The methotrexate analog bound to both wtDHFR and rDHFR-1 with a similar affinity. The measured K_d_s were 138.2 ± 9.9 nM for wtDHFR, and 194.5 ± 16.0 nM for rDHFR-1 (Fig. 3B). Additionally, rDHFR-1 did not show reducing activity towards dihydrofolate (DHF) (Supplemental Figure S2). NADPH binding to rDHFR-1 was also observed by ^15^N-^1^H TROSY spectra. Apo ^15^N-rDHFR-1 displayed good dispersion in the proton dimension from 7.1 to 9.5 ppm, indicating well-formed secondary structure. NADPH was added to the ^15^N-rDHFR-1 sample to a final ratio of 10:1 NADPH to ^15^N-rDHFR-1. The overlay of this spectrum onto the spectrum of apo ^15^N-rDHFR-1, revealed multiple chemical shift perturbations and the appearance of additional cross-peaks (Fig. 4). Overall, approximately 122 cross-peaks were present in ^15^N-rDHFR-1 spectrum and approximately 148 in ^15^N-rDHFR-1: NADPH spectrum. The significant changes in TROSY spectrum upon addition of NADPH can be explained by binding of NADPH to rDHFR-1.

### Structure characterization

**Figure 5:**
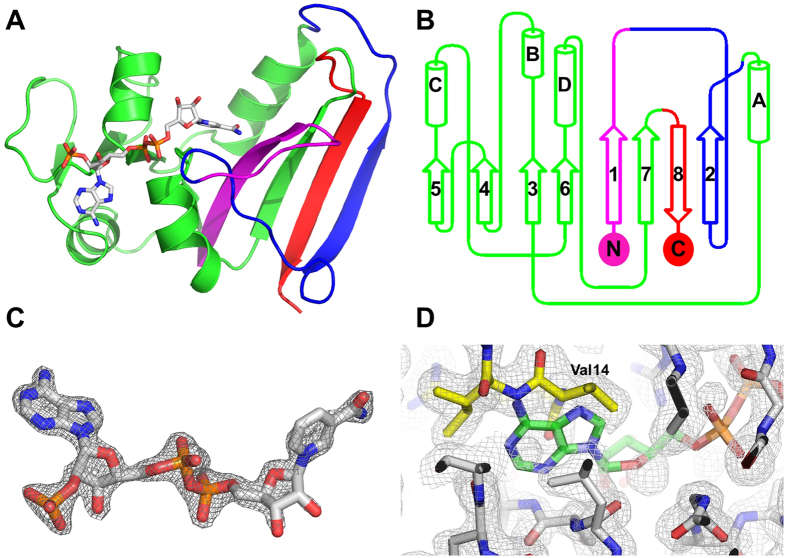
The structure (**A**) and topology (**B**) of the rethreaded DHFR. The secondary structural elements are labeled in (**B**). The order of the secondary structural elements changes from wtDHFR (Fig. 1B) due to the rethreading process. The color scheme used is the same as in Fig. 1. (**C**) Omit map contoured at 2.0σ around the NADPH molecule bound to the A chain. (**D**) Crystal packing prevents NADPH from binding to the B chain, which is shown with gray carbons. The neighboring molecule in the crystal is shown with yellow carbons. The location of the NADPH is obtained by superimposing the A chain on the B chain and is shown with green carbons in semi-transparent colors. The omit map contoured at 2.0σ around this region for the B chain is shown.

We determined the structure of rDHFR-1 to 1.60 Å (Fig. 5A). The space group was identified to be C222_1_, and two molecules were found in the asymmetric unit. All three new loops were observed in the electron density for one of the molecules (chain A). In the second molecule (chain B) two loops were ordered, and three residues had only partial density in the third loop. All new loops made the designed connections and the rethreaded protein maintained the overall core structure of the native protein, except for the sites of the alternate loops, as expected. Therefore, the four fragments were successfully reconnected as anticipated (Fig. 5A,B), without changing the core of the protein. After structural alignment with the wtDHFR, an RMSD of 0.88 Å over 116 residues was obtained for C_α_ atoms. These 116 residues represent mainly the undisturbed core of the protein (corresponding to most of fragments 1 and 3). In comparison, the A and B molecules of rDHFR-1 are more similar with an overall RMSD of 0.61 Å over almost the entire protein (145 residues).

**Figure 6:**
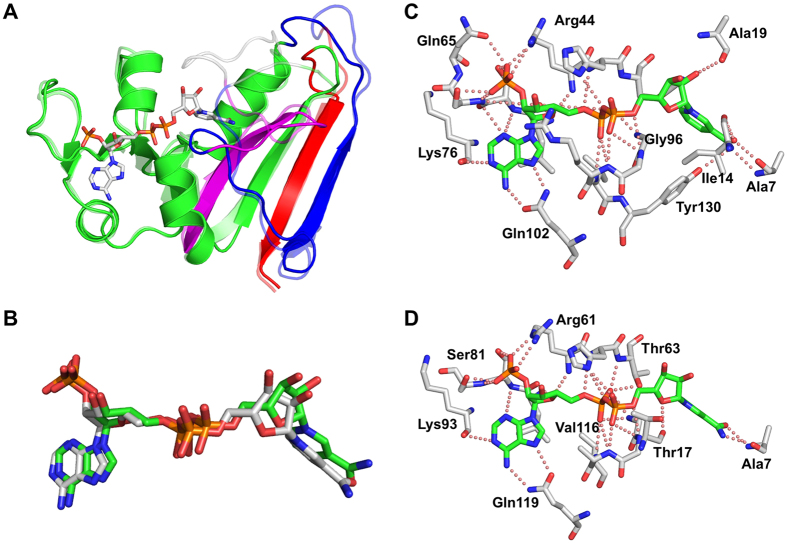
(**A**) Superposition of the wtDHFR (semi-transparent) and the A molecule of rDHFR-1. The NADPH molecule is depicted. The color scheme used is the same as in Fig. 1. (**B**) Comparison of the NADPH molecules shows larger deviations at the nicotinic ring. The NADPH from the wtDHFR structure is shown with green carbons. The NADPH hydrogen bonding networks are shown in (**C**) for wtDHFR (PDB code 4P66) and in (**D**) for rDHFR-1.

We found that the protein was still able to bind NADPH. One NADPH molecule was bound to the A chain (Fig. 5A,C), but none to the B chain (Fig. 5D). Interestingly, no NADPH was added to the crystallization trials, and the NADPH observed in the structure is apparently a result of co-purification. A part of the NADPH binding site in the B molecule was observed to be involved in crystal contacts and thus this site was not available for binding NADPH (Fig. 5D). This may explain why only one NADPH molecule is observed. The largest deviations between the A and B molecules were found near the NADPH binding site as well as at some of the newly introduced loops as shown in supplemental Figure S3. The maximum deviation is 2.0 Å at residue Gly-84, the next largest deviation is 1.4 Å at position Gly-68 (Supplemental Figure S3). Therefore, the two molecules are overall highly similar. Comparison between rDHFR-1 and wtDHFR gives an RMSD of 0.92 Å for all the atoms of the NADPH molecule. A higher RMSD of 1.4 Å is found over the nine atoms that make up the nicotinic ring portion of the NADPH. Although, the exact reason for the loss of activity is not determined, the movement of the NADPH, and specifically the motion of nicotinic ring is a likely cause (Fig. 6). For both rDHFR-1 and wtDHFR the number of hydrogen bonds between DHFR and NADPH is about the same (Table S2). However, in the case of rDHFR-1 there are fewer hydrogen bonds around the nicotinic ring (Fig. 6C,D).

### Loop structures

**Figure 7:**
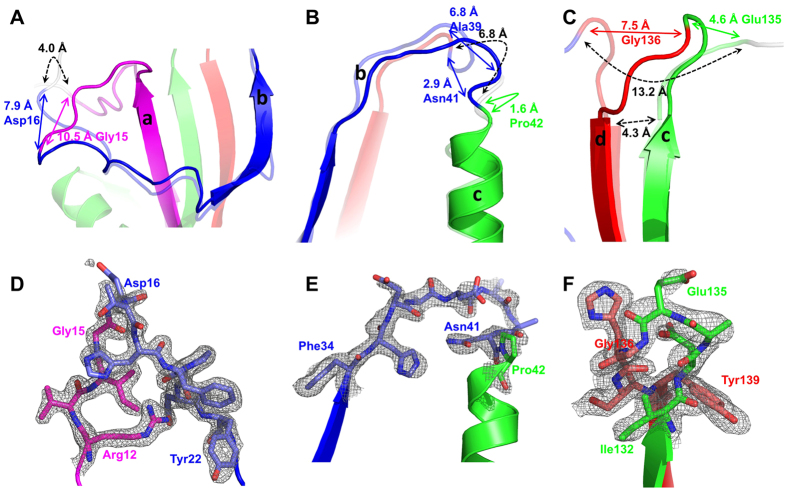
Superposition of rDHFR-1 and wtDHFR shows changes in the structure at the sites of the three new connections. The color scheme is the same as in Fig. 1, and the wtDHFR structure is shown as semi-transparent. The four fragments are labeled. The distances for the largest structural deviations are shown in colored arrows. The original distances between the fragments are also shown in black dashed lines. (**A**) Gly15 moves 10.5 Å, and Asp16 moves 7.9 Å. (**B**) Pro42 moves 1.6 Å, and Ala39 moves 6.8 Å. In this case the largest change is not at incision point at Asn41, which moves 2.9 Å. (**C**) Gly136 moves 7.5 Å, and Glu135 moves 4.6 Å. Although the distance between the incision points appears large (13.2 Å), the distance between the termini of the β-strands is only 4.3 Å (dashed black arrow). The residue numbers in this figure are based on rDHFR-1. Omit maps contoured at 2.0σ around the three incision points for the A chain are shown in (**D**–**F**).

Direct structural comparison between rDHFR-1 and wtDHFR may be difficult to interpret due to the change in the sizes of the loops and the fact that they are connected to different secondary structural elements. Nonetheless, the largest changes do occur at these modified sites. Upon rethreading, the order of the secondary structural elements changes (Figs 1A and 5B). Here we use the nomenclature for the wtDHFR as we describe the structural changes. The first link connects β strand-1 from fragment [a] to β strand-7 in fragment [b]. A long loop follows β strand-1 connecting it to helix-A in the wild type protein. Similarly, β strand-7 is preceded by a long loop in the native protein. In both cases, breaks were introduced within these long loops leaving behind a number of potentially flexible loop residues (Figs 1 and 2). Six residues on the side of fragment [a] moved more than 1 Å, with the largest shift being 10.5 Å at Gly-15. On the other side, five residues at the beginning of fragment [b] shifted more than 1 Å as well, with the largest change of 7.9 Å at position Asp-16 (Fig. 7A). The second link connects β strand-7 to helix-A. A break is introduced before Pro-25 (Pro-42 in rDHFR-1) with only one residue preceding helix-A, and another break in introduced leaving several loop residues past β strand-7. Thus, most of the connecting loop residues come from the segment that follows β strand-7. Indeed, only Pro-42 moves more than 1 Å, on the helix side of the new link. On the other side, six residues following β strand-7 move more than 1 Å, with the largest change of 6.8 Å at Ala-39. A smaller shift of 2.9 Å is observed at the incision point at Asn-41 (Fig. 7B). The third link connects β strand-6 to β strand-8. Although the distance between the incision points in this case appears to be large (13.2 Å), the distance between the termini of the β-strands 6 and 8 (residues 115 and 151) is only 4.3 Å. β strand-6 ends at residue 115. Residues Asp-116, Ala-117, Glu-118, and Gly-121 were included past β strand-6. Another break is introduced in the loop that precedes β strand-8. Three residues in fragment [c] and four residues in fragment [d] moved more than 1 Å (Fig. 7C), with the largest shift being 4.6 Å at Glu-135 and 7.5 Å at Gly-136. In all three cases, the loops proved to be flexible and able to adjust to the new connections as expected. The electron density around the incision points is shown in Fig. 7D–F. A total of 24 residues moved more than 1 Å with nine residues making substantial shifts of more than 4 Å. Residues that belonged to secondary structural elements in wtDHFR (1RX9)^24^ have not moved significantly (less than 1 Å). β strand-7 appeared longer in rDHFR-1 than in wtDHFR (1RX9), but this structural change has been observed in other wtDHFR structures as well. The B-factors at the incision points is slightly higher than the overall average B-factor, and is in range of what is observed for other loops (Supplementary Figure S4). The average B-factors are 30.58 Å^2^ for the A chain, 33.78 Å^2^ for the B chain, 37.63 Å^2^ and 46.68 Å^2^ at the incision points for the A and B chains respectively.

### Structure predictions

**Figure 8:**
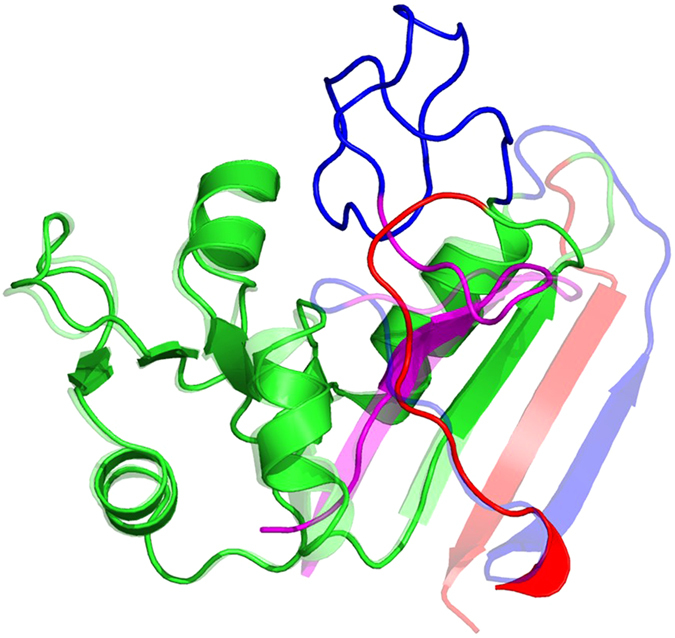
Structural alignment between rDHFR-1 and the predicted structure from the Phyre2 server. The structure of rDHFR-1 is shown as semi-transparent. The color scheme is the same as in Fig. 1. Fragment [a], in magenta, is predicted properly. However, fragment [b] in blue is misplaced in its entirety. Fragment [c], in green, is predicted properly. Lastly, fragment [d], in red, is misplaced, and threaded over where the [b] fragment is supposed to be.

We tested a number of structure prediction programs including Phyre2^32^, I-TASSER^33^, Muster^34^, Raptor-X^35^, and the Robetta server^36^. In all these cases the core 115 residues were predicted properly out of 149. Threading is performed in a linear sequential manner, thus fragment [a] (the first 15 amino acids) is predicted properly, however fragment [b] (Fig. 8 in blue) is misplaced in its entirety. Fragment [c] is predicted properly, and fragment [d] is threaded over where fragment [b] is supposed to be (Fig. 8). Threading programs do not recognize that a rethreading process was performed. The Dali^41^ server identifies wtDHFR as the closest structure homolog to rDHFR-1, however it only aligns 121 residues. Similar to the threading programs DALI does not align the [b] fragment properly.

## Discussion

The requirement of the simultaneity of the rethreading process may be one of the main obstacles for success. However, here we have demonstrated that indeed protein rethreading can be implemented successfully, even when loop sequences are not optimized. In this case, we tested if protein rethreading can be carried out with minimal changes to the native amino acid sequence, thus loop selection was limited to residues already present at the incision sites. This highlights two points. First, loops can offer flexibility and do not need to be perfectly selected in order for a protein rethreading procedure to work. Second, with improved loop design a wider range of possible connections can be prepared.

The rethreading performed here is similar to what has been described as a triple-point chain switching^42^. However, the triple point switching requires all three crossover points to be proximal, and thus can be a rare occurrence. We have shown here that the crossover points do not need to be proximal. Trimming some of the loops allowed the crossover points to be distant. Furthermore, by relying on the loops to be flexible and able to adjust their structures in accordance with the new linkages, it became possible for the crossover points to be even more distant.

It has been shown that new protein folds can be designed de novo computationally^2^, however for larger proteins this can be challenging and time consuming. Here, we have shown that protein rethreading can be a practical and quick approach to produce novel protein topologies, if not folds. Importantly, rethreading is not limited by the size of the target protein, and larger proteins may indeed offer a greater number of possible incision points suitable for rethreading. It has been observed that naturally occurring proteins can have similar folds but different connectivity. This has been described as a form of multiple loop permutations (MLP)^20^. Although MLP and triple-point chain switching^42^ have been computationally analyzed previously, rDHFR-1 represents the first experimental demonstration of protein rethreading. Proteins related by MLP are considered to be distinct folds^20^, thus rDHFR-1 can be considered a new fold.

It is well accepted that protein cores play essential roles in determining their structures. The peptide chain takes a certain path through the 3D space in order for a protein core to form properly. The path taken by the peptide chain depends on the protein fold. Here we demonstrated that it is possible to alter the path of the peptide chain, at certain junctions, yet maintain the core structure of the protein with minimal changes. We have found that structure prediction programs, including the Robetta server do not predict the entire structure correctly (Fig. 8). The predictions are accurate for most of fragments [a,c], but miss fragments [b,d] due to the new connections, which are not readily recognized by the prediction programs. Additionally, it is well recognized that proteins of similar sequences will adopt the same overall structures. However, sequence comparisons are typically carried out in a linear fashion. Yet, we know that residues can be very close in 3D space, although distant in sequence. Here we demonstrated that it is possible to alter the path of the peptide chain by connecting residues that are close in space, but distant in sequence while maintaining the overall structure of the protein intact. The inclusion of this 3D information into sequence alignment or structure prediction programs might allow for the recognition of similarities between proteins that would otherwise remain unnoticed.

Circular permutations have proven very useful, thus it is reasonable to expect that protein rethreading can be utilized in a similarly beneficial manner. However, few distinctions are apparent. The extent of the potential protein space that can be attained by multiple loop permutations has been shown to be large. The application of MLP to 2936 SCOP^43^ domains, resulted in the identification of 2843 new structures^20^. Therefore, on average about one new domain structure can be designed for every SCOP domain with this approach. Although the rethreading process performed here is analogous to MLP, rethreading is not limited to swapping loops. Rethreading can be more expansive than MLP in the following ways: 1- breaks within secondary structural elements can be introduced, 2- a number of residues may be removed as shown with rDHFR-1 which is 12 residues shorter than wtDHFR, 3- the original N and C termini can be used as connection points to a newly introduced terminus, and 4-rethreading can be used on multi-domain proteins or to fuse protein complexes^25^. Therefore, the potential number of protein architectures that can be engineered using rethreading is likely to be vast.

The success of the rethreading experiment suggests that the sequence/structure relationship can be expanded substantially. The sequence to structure relationship is interrelated. Indeed, computational methods, such as the rosetta program that do well in protein design^2^,^3^,^4^,^5^,^6^ typically also do well in structure prediction^44^. Even though rethreading DHFR was mainly a protein design process, it can have implications on the field of structure prediction. Sequence alignment can identify proteins that adopt the same overall structure. Threading helps increase the number of sequences recognized to belong to the same fold^18^. Here we have shown that by careful rearrangement of a protein sequence, a modified structure can be generated. Thus, the success of rethreading demonstrates that the sequence to structure relationship can be expanded further, and that a larger array of sequences can be expected to fit into a set of carefully altered structures.

## Additional Information

**How to cite this article**: Agah, S. *et al*. Protein rethreading: A novel approach to protein design. *Sci. Rep.* **6**, 26847; doi: 10.1038/srep26847 (2016).

## Supplementary Material

Supplementary Information

## Figures and Tables

**Table 1:** Data collection and refinement statistics*.

Space group	C222_**1**_
Resolution range (Å)	40.0–1.60 (1.63–1.60)
Cell a, b, c (Å)	44.84, 120.05, 111.19
alpha, beta, gamma (°)	90, 90, 90
Completeness (%)	95.3 (63.4)†
Redundancy	6.3 (2.7)
Rmerge	7.3 (27.1)
I/ Sigma	25.5 (4.0)
cc 1/2 (1.6–1.63 Å)	0.919
Number of unique reflections measured	38155
Number of reflections used in refinement	36214
Number of reflections used in Rfree set	1911
Rwork/Rfree (%)	18.26/21.34 (22.3/27.2)
Bonds RMS (Å)	0.024
Angles RMS (°)	2.279
Protein atoms	2318
Solvent atoms	153
Mean B factor (Å^2^)	31.59

^*^Numbers in parenthesis reflect the highest resolution shell (1.60–1.63 Å).

^†^Completion for each resolution shell are shown in supplemental table S1.
