# Supplementary material for: Protein rethreading: A novel approach to protein design
Source: Sci Rep. 2016 May 27;6:26847. doi: 10.1038/srep26847 (PMC4882587; doi:10.1038/srep26847)
Supplement: Supplementary Information [file srep26847-s1.doc]

**Protein rethreading:**

**A novel approach to protein design**

Sayeh Agah, Sandra Poulos, Austin Yu, Iga Kucharska, Salem Faham


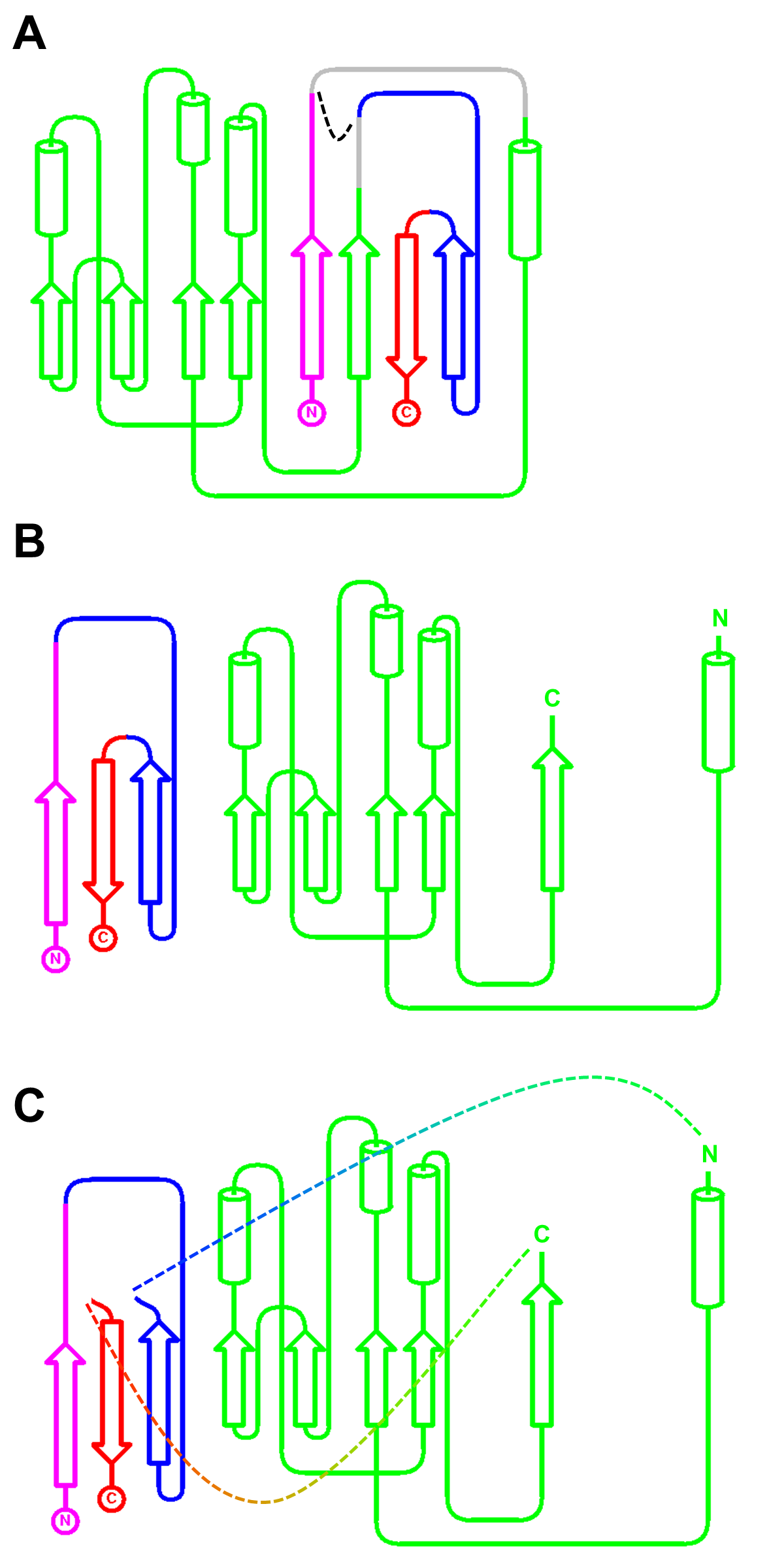


**Supplemental Figure S1.** The rethreading process cannot be performed in a stepwise manner. Panel (A) shows that in order to form one new link (between the magenta and blue fragments), two bonds need to be broken. As a result, as shown in (B), the protein ends up in two separate polypeptide chains. The (C) panel demonstrates how it is possible to reconnect the two polypeptide chains into a single connected chain. An additional break is introduced between the blue and red fragments, which now can be linked to the free termini of the green fragment.


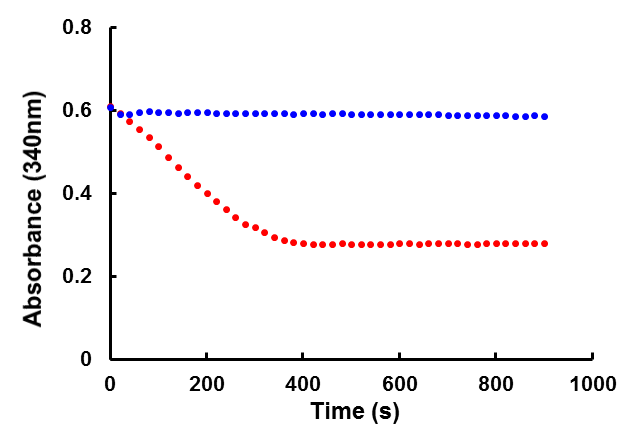


**Supplemental Figure S2.** Dihydrofolate reduction activity assay. Comparison of the activity of wtDHFR (red) to rDHFR-1 (blue). A tenfold higher concentration was used in the case of rDHFR-1 (1 µM), compared to wtDHFR (100nM).


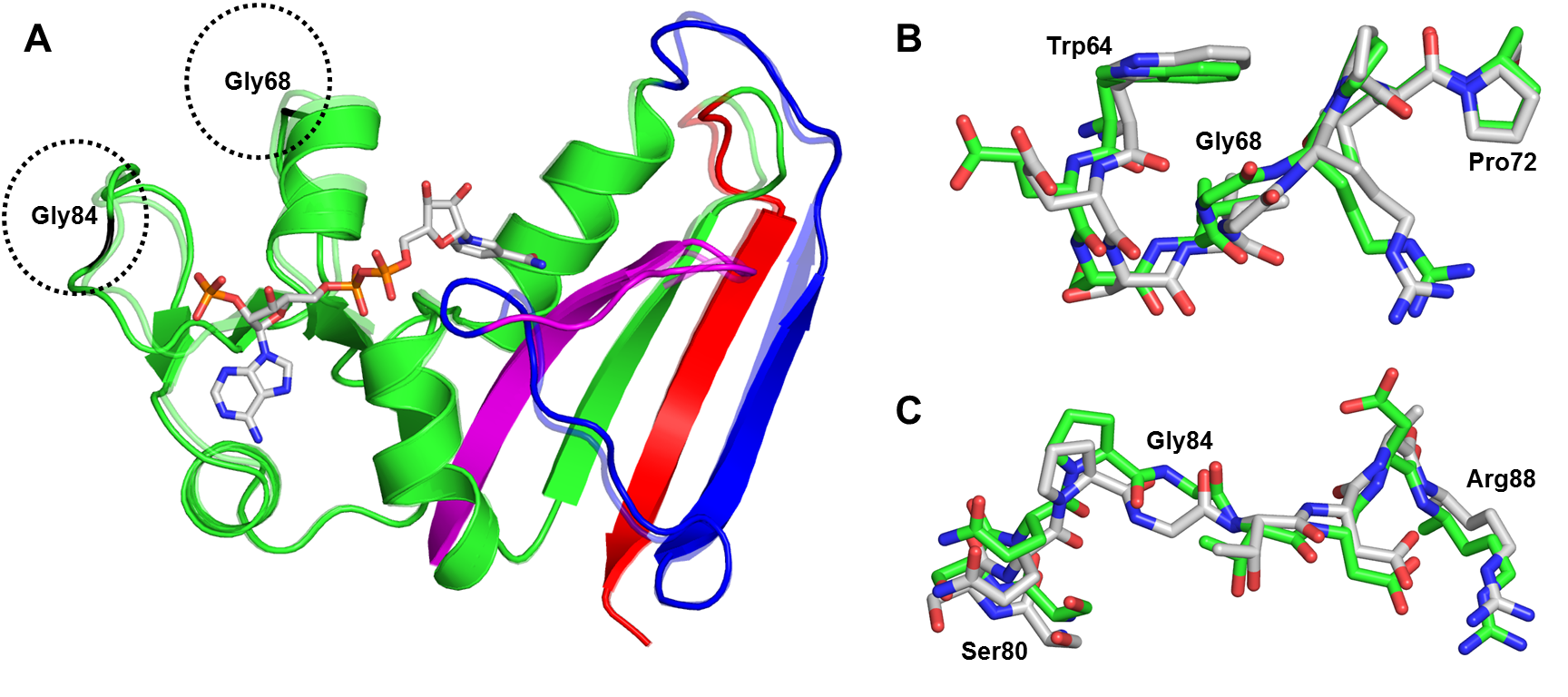


**Supplemental Figure S3.** Superposition between the A and B chains. (A) Only the A chain was observed to bind to an NADPH molecule. The B chain is shown in semi-transparent colors. The color scheme used is the same as in Figure 1. The largest deviations between the A and B chains are found at positions Gly-84 and Gly-68. Stick representation of the region around residues 68 and 84 are shown in (B) and (C), respectively. For both (B) and (C) the carbon atoms colored in green for the A chain and in gray for the B chain.


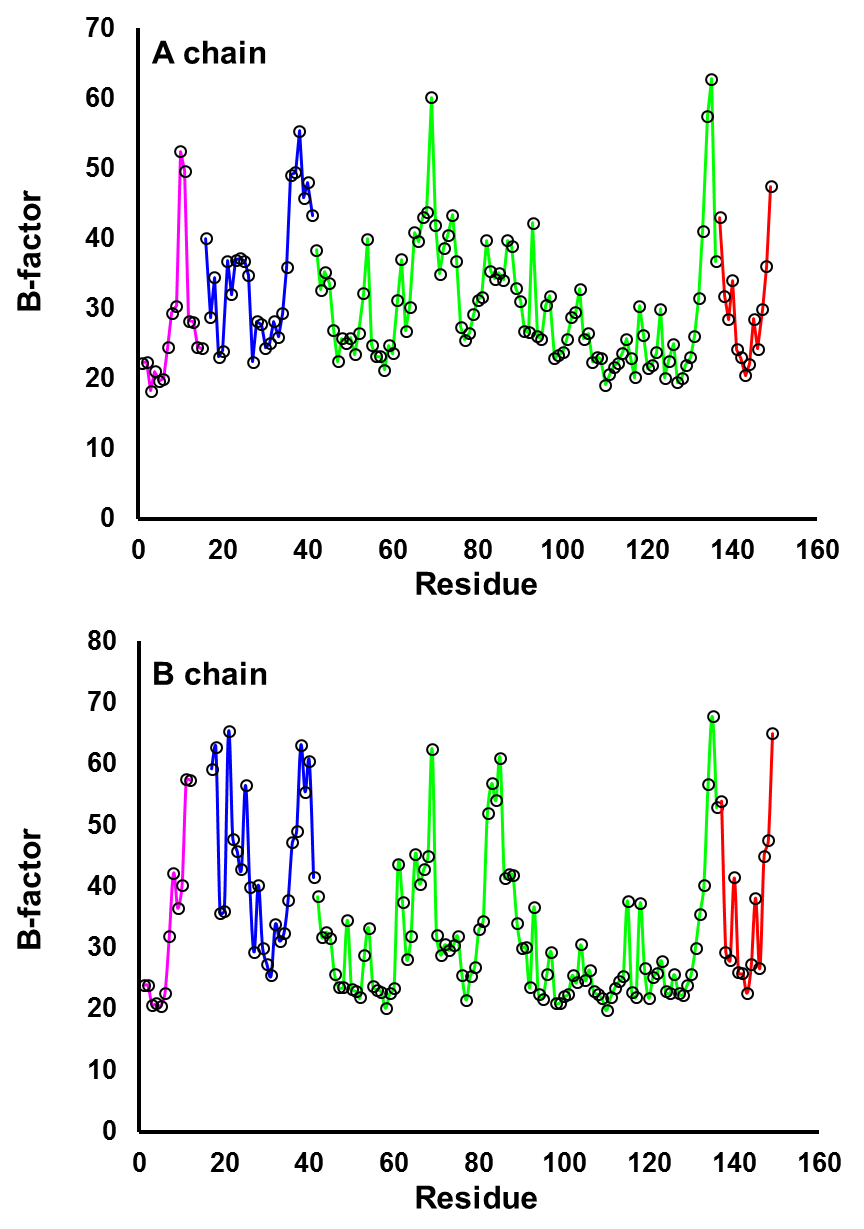


**Supplemental Figure S4.** B factors plots for the A and B chains. Each fragment is colored with the same scheme used in Figure 1.

**Supplemental Table S1.** Data completion per resolution shell.

| Resolution limits | |  |
| --- | --- | --- |
| Low (Å) | High (Å) | Completion (%) |
| 40.0 | 4.34 | 95.1 |
| 4.34 | 3.45 | 99.6 |
| 3.45 | 3.01 | 100 |
| 3.01 | 2.74 | 100 |
| 2.74 | 2.54 | 100 |
| 2.54 | 2.39 | 100 |
| 2.39 | 2.27 | 100 |
| 2.27 | 2.17 | 100 |
| 2.17 | 2.09 | 100 |
| 2.09 | 2.02 | 100 |
| 2.02 | 1.95 | 100 |
| 1.95 | 1.90 | 100 |
| 1.90 | 1.85 | 100 |
| 1.85 | 1.80 | 100 |
| 1.80 | 1.76 | 99.8 |
| 1.76 | 1.72 | 97.4 |
| 1.72 | 1.69 | 92.2 |
| 1.69 | 1.66 | 84.0 |
| 1.66 | 1.63 | 72.4 |
| 1.63 | 1.60 | 63.4 |
| 40.0 | 1.60 | 95.3 |

**Supplemental Table S2.** Comparison of the hydrogen bonding networks for NADPH. rDHFR-1 is shown in red, and wtDHFR is shown in black and is based on PDB code 4P66. Atom names for the NADPH molecule are the same as in 4P66.

| NADPH Atom | Protein Atom | Residue | Distance Å | Protein Atom | Residue | Distance Å |
| --- | --- | --- | --- | --- | --- | --- |
|  |  |  |  |  |  |  |
| O1A | N | Gly113 | 3.19 |  |  |  |
|  | N | Arg115 | 3.37 | N | Arg98 | 3.39 |
|  | N | Val116 | 3.26 | N | Val99 | 3.14 |
| O4B | N | Arg61 | 3.25 | N | Arg44 | 3.21 |
|  | O | Leu79 | 3.33 | O | Leu62 | 3.37 |
| O5B | N | His62 | 3.38 | N | His45 | 3.46 |
| O1X | NE | Arg61 | 2.65 | NE | Arg44 | 2.59 |
|  | OG | Ser80 | 2.61 | OG | Ser63 | 2.64 |
|  |  |  |  | OE1 | Gln65 | 2.98 |
| O2X | N | Ser81 | 2.78 | N | Ser64 | 2.79 |
|  | OG | Ser81 | 2.54 | OG | Ser64 | 2.74 |
| O3X | NH2 | Arg61 | 2.86 | NH1 | Arg44 | 3.19 |
| N1A | O | Lys93 | 3.22 | O | Lys76 | 3.28 |
| N3A | N | Ser80 | 3.48 | N | Ser63 | 3.44 |
|  |  |  |  | N | Ser64 | 3.43 |
| N6A | NE2 | Gln119 | 2.94 | OE1 | Gln102 | 2.73 |
| N7A | OE1 | Gln119 | 2.98 | NE2 | Gln102 | 2.78 |
| O2A | N | Thr63 | 2.87 | N | Cys46 | 3.06 |
|  | N | Gly113 | 3.09 | N | Gly96 | 2.79 |
|  | OG1 | Thr63 | 2.79 |  |  |  |
| O1N | N | Gly114 | 3.38 | N | Gly97 | 3.35 |
|  | N | Arg115 | 2.82 | N | Arg98 | 2.94 |
| O2N | ND1 | His62 | 3.08 |  |  |  |
| O3 | ND1 | His62 | 3.25 | ND1 | His45 | 3.42 |
| O5D | N | Gly114 | 3.09 | N | Gly97 | 3.21 |
| O2D |  |  |  | O | Ala19 | 3.29 |
| O4D | O | Thr17 | 3.5 |  |  |  |
| O7N | N | Ala7 | 2.74 | O | Ala7 | 2.79 |
|  |  |  |  | O | Ile14 | 3.23 |
| N7N | O | Ala7 | 2.71 | N | Ala7 | 2.98 |
|  |  |  |  | OH | Tyr100 | 3.14 |
